# EFL Learner Engagement in Automatic Written Evaluation

**DOI:** 10.3389/fpsyg.2022.871707

**Published:** 2022-05-23

**Authors:** Huixia Fu, Xiaobin Liu

**Affiliations:** School of Foreign Studies, South China Normal University, Guangzhou, China

**Keywords:** automatic written evaluation (AWE), feedback, engagement, EFL learner, writing

## Abstract

Recent years have witnessed increasing popularity in the use of automatic written evaluation (AWE) in the writing context for its immediacy and high accessibility for EFL learners. Meanwhile, the effectiveness of the AWE tool in writing accuracy and ability is fully appreciated by the previous researchers. However, students' engagement in the revising process, key factors that mediate the uptake of feedback, and learning effect have not aroused much attention as expected. Thus, this review aimed to depict a broader picture of learners' behavioral, cognitive, and affective engagement in AWE feedback to bring a further understanding of how learners process the feedback and make the decision from a psychological perspective. Firstly, widely adopted targets in AWE research are discussed. Then, the definition of learner engagement and its constructs are presented based on existing research. After that, the link between AWE feedback and learner engagement has been taken into consideration. Finally, conclusions and suggestions are provided for insightful studies.

## Introduction

Feedback is perceived as an essential part of writing instruction. With the advent of technology, automatic written evaluation (AWE) has become an increasingly popular facilitator for learners to monitor their writing process and regulate their learning process. What sets AWE apart from traditional feedback is sophisticated error identification and timely feedback. Immediate feedback including global organization, language use, and mechanics is provided through the AWE system (Stevenson and Phakiti, 2019). However, feedback alone cannot directly bring a writing improvement., the effectiveness of feedback is significantly confined to how learners deal with it. Thus, what unlocks the benefits of it is student engagement with this response to work (Zhang and Hyland, 2018). An ever-expanding body of work concerning AWE feedback has demonstrated its effectiveness on learners' writing performance while scant evidence shows how learners perceive and engage in the AWE feedback during the writing process. Little has been told from the previous research on how learners adjust their metacognitive skills to internalize the AWE feedback and improve their writing. It is suggested that a deeper exploration of learners' cognitive and affective engagement is pressing (Stevenson and Phakiti, 2019). To figure out what may be the factors restraining the potential of AWE feedback, this paper shifts the attention toward the complex interplay of learner engagement with AWE rather than the effectiveness and final product of the work. From this perspective, the following questions guide this review.

[1] What are the widely adopted targets in AWE research over years (2017–2021)?

[2] How does the existing research reveal learner engagement and relate it to corrective feedback?

[3] How does the research uncover learner engagement in AWE?

In this review, AWE and learner engagement have been discussed. Conclusion and suggestions for future study are provided.

## Literature Review

### Automatic Written Evaluation

Artificial intelligence and Natural Language Processing (NLP) system led to the emergence of AWE, a web-based system for automatic scoring and feedback on written work (Shermis et al., 2013). Within this umbrella term, automatic written corrective feedback (AWCF) refers to the use of the AWE tool to provide its users with feedback based on the grammatical lapses in their written work. A wide range of affordances for L2 writing favored by AWE has been explored and acknowledged. AWE empowers learners to get feedback promptly based on their draft and positively impacts students' revision practices (Link et al., 2020). Writing improvement can be highlighted for the immediacy and directness of AWE in offering nuance feedback (Shang, 2019). By leading support to peer assessment, AWE shows great potential for the cultivation of learners' positive mentality (Yao et al., 2021). Thus, AWE can be universally considered as an ideal feedback provider and writing helper.

In terms of psychological perspective, L2 learners who are exposed to AWE during the writing process achieved a desirable result concerning the L2 learners' overall writing accuracy due to the affordance of the AWE tool to “promote noticing, provide an adaptive metalinguistic explanation, and engage students in self-directed learning” (Barrot, 2021). Learners' development in accuracy could be greatly accelerated by cognitive processing with AWCF, including noticing, understanding and evaluation. Furthermore, learners are more likely to internalize the knowledge from AWE feedback and store it in their long-term memory for later retrieval, which also confirms the long-time effect of AWE on learning (Link et al., 2020). During this process, the metacognitive process plays a pivot role in successful revision and true learning, in which automatic feedback enables learners to notice a gap between their knowledge of the L2 and the accurate use of language to consciously adjust their language use. The interaction between the learners and the AWE feedback reveals the complexity and non-linearity of the revision process in which students act as active and autonomous agents to filtrate the AWE feedback, rather than blind recipients (Bai and Hu, 2017). Stevenson and Phakiti (2019) deemed it promising for AWE feedback to become increasingly sophisticated and much more pervasive.

### Learner Engagement

Engagement is conceptualized as “energized, directed, and sustained action, or the observable qualities of students' actual interactions with academic tasks.” (Skinner and Pitzer, 2012, p. 24). Learners' meaningful participation is significantly addressed in the learning process. Ellis (2010) favored learners' engagement with corrective feedback (CF) as an action construct that captures its behavioral, cognitive, and emotional dimensions. Han and Hyland (2015) further explained how these engagements work in the face of feedback. Cognitive engagement in CF is concerned with learners' use of cognitive strategy (e.g., monitoring and evaluating). Behavioral engagement encompasses implementing this outcome of processing activity via revision strategies while affectively engaged calls for the positive and negative responses to the feedback.

Engagement embraces an intrinsically complicated interplay of various cognitive, and psychological factors. It concentrates on how resources, effort, and time are allocated to different tasks. What the teachers can observe in the writing process is learners' acceptance and reluctance toward the feedback. Although behavioral engagement is most likely to be the observable indicator of writing performance, emotion is fundamentally the underlying driver of high-quality learning (Skinner et al., 2008). High engagement is linked to a positive outcome (Hiver et al., 2021b), indicating active involvement and meaningful participation. Concerning the degree of engagement, it varies from learners' use of behavioral, affective, and cognitive strategy, proficiency to belief, which also results in different responses to the feedback and revision outcome (Han, 2017; Zhang and Hyland, 2018).

### The Link Between AWE Feedback and Learner Engagement

Learning takes place only when learners are truly involved (Hiver et al., 2021a). Learning effect in AWE feedback is worthy of further consideration of learners' involvement and engagement. How to perceive and deal with feedback from AWE is not simply an understanding and revising operation but more of mental activities. According to Koltovskaia's (2020) framework, engagement with AWCF encompasses three interrelated components, behavioral, cognitive, and affective engagement. Behavioral engagement with AWCF involved the time allocation, operation, and strategies of revision. Cognitive engagement is perceived as learners' use of metacognitive and cognitive strategies to process the feedback. Affective engagement with AWCF involved students' emotions and attitudes toward the AWCF feedback. Behavioral engagement alone is less likely to guide successful revisions unless the accurate AWCF is accepted (Koltovskaia, 2020). It seems that students did do the revision but what matters is whether they are cognitively or affectively engaged with the AWE feedback for deeper learning or lasting effect. How learners process feedback, make judgments, and do revise remains unclear. Therefore, more metacognitive evidence is needed to capture an increasingly comprehensive picture of engagement that helps learners notice, make the decision and transfer to other contexts.

Tsao et al. (2021) claimed that learner engagement with written corrective feedback (WCF) occupies a more central place in predicting writing performance than intrinsic motivation, which remarkably mediates the causal relation between intrinsic motivation and writing performance. With the frequent use of metacognitive and cognitive operations, extensive cognitive engagement with AWCF enables learners to make the evaluation and selective incorporation (Koltovskaia, 2020). As a joint result of the interaction of three engagements, learners tend to make critical analyses to prevent over-dependence. Based on a case study, Zhang (2017) reported a positive impact of computer-mediated feedback on writing when investigating how Chinese EFL learners are behaviorally, cognitively, and affectively engaged. Zhang and Hyland (2018) revealed that AWE feedback promoted a more autonomous engagement. The learner plays a dominating role in their learning process, which encourages autonomous awareness and self-regulation. Lee (2020) noted that learners engaged themselves in writing through the adoption of a variety of composing and problem-solving strategies including editing based on the automated content feedback system. AWE undoubtedly provides a great amount of feedback, but the uptake rate is highly associated with learners' selective utilization, which reveals a dynamic engagement with automated feedback (Bai and Hu, 2017; Tian and Zhou, 2020). Individual factors, as well as context factors, mediate learner engagement. Higher proficiency learners are inclined to embrace successful revision due to their linguistic competency and cognitive strategy (Zhang, 2020). Additionally, human-automation trust is another concern to explain engagement with AWE feedback accuracy. Factors related to AWE itself including accuracy and feedback explicitness lead to the variation in engagement (Ranalli, 2021).

## Methodology

**Figure 1:**
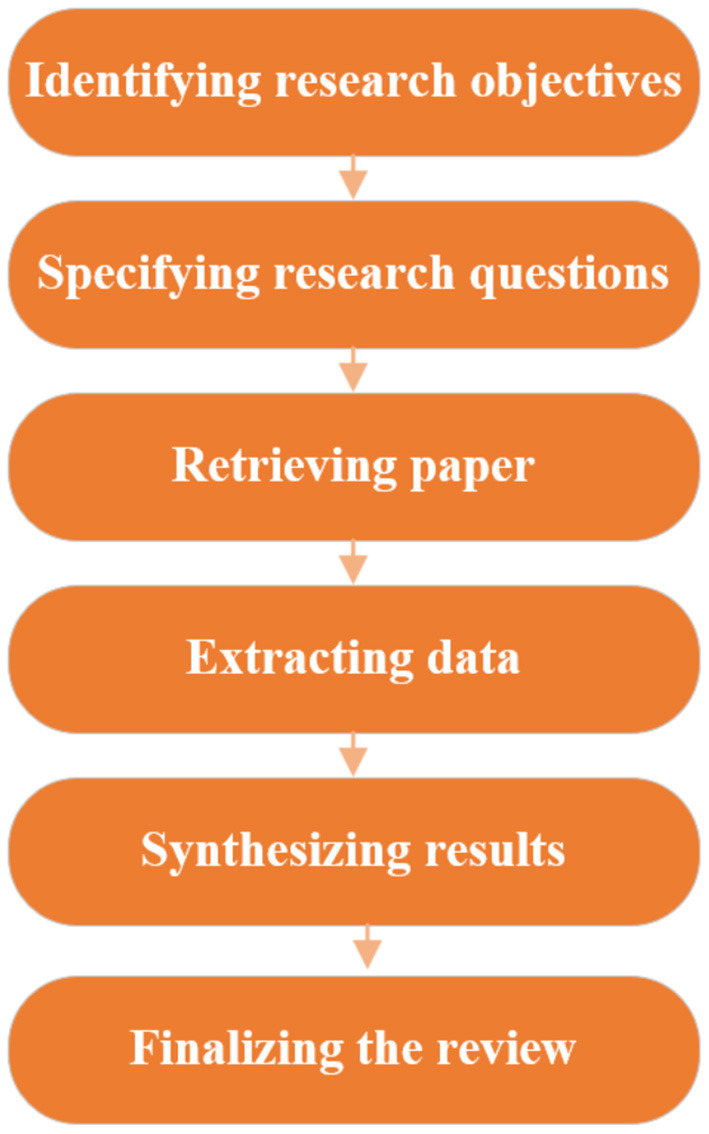
Procedures to conduct this review.

To address the research questions and yield valuable insights into further study, this paper includes the review targeting AWE and engagement as well as their links. Keywords used in the retrieval consist of three categories (i.e., feedback, engagement, and writing), including feedback, evaluation, corrective feedback, automatic written corrective feedback, automatic written evaluation, computer-generated feedback, technology-assisted feedback, engagement, revision, response, perception, and writing. This review selected the core collection of research and highly cited papers throughout the database to capture the current trend, including Web of Science, Taylor and Francis Online, SAGE, Springer, Elsevier, Wiley Online Library, Frontiers Media SA, Cambridge University Press, and Oxford University Press. Notably, the selected articles for analysis are empirical studies, comprising quantitative and qualitative or mixed studies, published in SSCI and SCI journals from 2017 to 2021, but articles that did not focus on AWE and engagement in language learning are excluded. The review articles and books in this paper are used to define the key concepts and add up to the finding of this result but are excluded from the analysis. Additionally, the selection of articles concentrates on the research topics, perspectives, and methodology of the studies with little concern about the participants, the types of AWE tools, and direct or indirect feedback. Nevertheless, engagement in other sources of feedback is not the primary research focus but their combination or comparison with AWE is taken into consideration. Moreover, the data collections start from the abstract, methodology to conclusions within these studies. And then similarities and differences are extracted to synthesize the result. The review conduct is as follows (Ishaq et al., 2021) (Figure 1).

## Result

**Figure 2:**
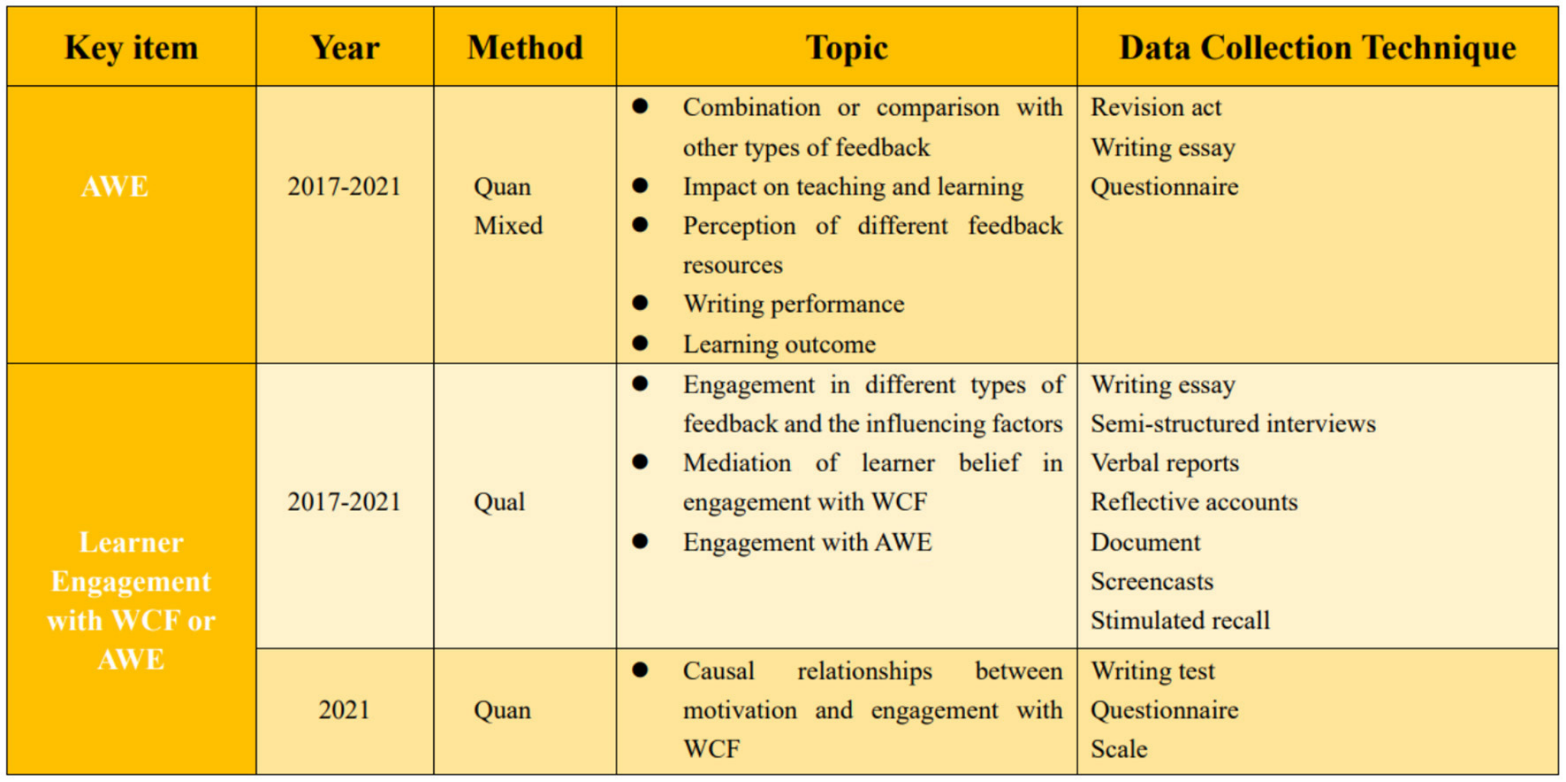
Inclusion of the empirical studies in this review.

As is shown in the literature review and Figure 2, the effectiveness of AWE in writing accuracy is widely discussed. Most research on how learners engage with WCF or AWE is qualitatively conducted with interviews and self-reports. As a powerful instructional, AWE deserves deeper exploration with richer data resources to identify how learners interact with its feedback to facilitate their learning. A trend is suggested to adopt learners' perspectives to stress their voice in learning engagement.

To better interpret the complex construct of engagement, its choice of measurement is of paramount importance for the invisible and implicit dimensions to predict the variance of degree, such as motivation (Skinner and Pitzer, 2012). The use of multiple measurements and complementary data sources are encouraged to provide additional explanations for the existing results for learner engagement (Hiver et al., 2021b; Zhou et al., 2021). Additionally, behavioral, affective, and cognitive responses are simultaneously at work, dynamically interacting with each other. The developmental changes should be observed through an ongoing process. Besides, engagement can also be investigated as an intervening variable between the provision of AWE feedback and writing improvement, which helps to further discover the underlying effect by establishing the structural equation modeling.

Methodologically, there is a lack of mixed studies used to trace and detect how learners' engagement develops within the learning process. Although engagement with AWE gradually has turned in the spotlight, much research was conducted with a qualitative method by analyzing learners' words from the interview and questionnaire (Figure 2). Meanwhile, case study occupies a high proportion of existing research. To obtain an in-depth understanding of learning engagement with feedback, case studies involving students with different language proficiency were employed to analyze students' engagement with AWCF qualitatively in the use of stimulated recall, semi-structured interviews and retrospective verbal reports, reflective journals, etc. (Han, 2017; Koltovskaia, 2020; Zhang, 2020; Ranalli, 2021). Although these methods opt to figure out how and why learners engage with WCF, they can serve as a complementary perspective to support quantitative methods for deeper understanding. In this case, more scientific instruments and a larger scale can be taken into consideration to enhance generalization.

## Conclusions and Suggestions

This paper reviewed the research on AWE and learner engagement with AWE from their different methods and perspectives. Regarding the first research question, our results showed that previous research pinpoints AWE plays a role in the writing performance and learning outcome by quantitively analyzing the writing drafts. Meanwhile, closer attention has also been increasingly given to the learners' acceptance and cognitive internalization of the feedback. Concerning the second question, the result suggested that not only the feedback provider, but engagement should be highly valued. A greater concern is placed on psychological and mental factors contributing to high engagement. More indirect features (e.g., motivation, anxiety) are desirable for measurement to reveal the dynamic process of engagement in the face of feedback. To answer the third question, the results demonstrated the interconnectedness of three constructs in engagement with AWE and the significance of learners' response to AWE for its full play. However, the engagement of the particular individuals is mostly qualitatively explored in case studies. Nevertheless, a mixed study is extensively welcomed to offer cogent evidence (Moser, 2020). Multiple methods can facilitate the elicitation of learners' thoughts to present a finer-grained picture of learner engagement (Shi, 2021).

In line with the existing research, this article revealed that simply providing AWE feedback will not bring forth continuous improvement. More consideration can be placed on learners' engagement (Reynolds et al., 2021). The combination of three engagement types renders learners' active utilization of their psychological strategy to maximize the effect of the AWE. Notably, learners' engagement in feedback should not be taken for granted (Nguyen, 2021). Belief in sources of feedback and trust systems can inevitably influence their involvement. A possible solution for insufficient engagement is scaffolding by the teachers. Teachers' support can greatly mediate learners' experience in the AWE (Jiang et al., 2020) and build up students' trust system to accept AWE. Learners can be guided to be cognitively engaged by questioning and analyzing the AWE feedback critically so that productive engagement can be achieved by accessible validation (Koltovskaia, 2020). Furthermore, teachers' feedback can act as a powerful supplement. Teachers' support should be lent to regulate learners' emotional responses, increase their motivation, and use cognitive strategies (Zhang, 2020). Besides, it is essential to view learner engagement from the ecological perspective instead of static or isolated by taking the individual, instructors, and contextual factors into consideration.

To advance the research on engagement, more sophisticated techniques (e.g., inputlog) together with eye-tracking recordings and thinking aloud protocols can help to uncover the underlying involvement and cognitive processing (Leijten and Van Waes, 2013). When exposed to AWE during the revision and rewriting process, learners' cognitive and affective engagement can be better visualized by their revision behaviors and strategies through multiple techniques.

In a nutshell, AWE is becoming a promising tool for its constant updating, and learners' engagement with AWE is expected to be deeply investigated to fully fulfill the value of AWE in learning. In addition, this review potentially contributes to a broader understanding of this domain.

## Author Contributions

XL: conceptualization, supervision, project administration, and funding acquisition. HF and XL: methodology and writing—review and editing. HF: writing—original draft preparation. Both authors have read and agreed to the published version of the manuscript.

## Funding

This research was supported by Center for Language Cognition and Assessment, Guangdong, China.

## Conflict of Interest

The authors declare that the research was conducted in the absence of any commercial or financial relationships that could be construed as a potential conflict of interest.

## Publisher's Note

All claims expressed in this article are solely those of the authors and do not necessarily represent those of their affiliated organizations, or those of the publisher, the editors and the reviewers. Any product that may be evaluated in this article, or claim that may be made by its manufacturer, is not guaranteed or endorsed by the publisher.
